# Experimental design of 4-point supported belt robot for sanding large convex surfaces

**DOI:** 10.1038/s41598-024-56650-w

**Published:** 2024-03-22

**Authors:** Hanbom Kim, Hongjoo Jin, Woojae Lee, SeungHeon Chae, Taegyun Kim, TaeWon Seo

**Affiliations:** 1 School of Mechanical Engineering, Hanyang University, Seoul, 04763 Republic of Korea; 2 Mechanical Engineering, Yeungnam University, Gyeongsan, 38641 Republic of Korea

**Keywords:** Engineering, Mechanical engineering

## Abstract

In general, sanding robots that move as if drawing a line along a surface are mainly used when sanding objects with a large area; however, they require a long working time, and it is difficult to secure a uniform sanded area. This study focuses on large-area sanding robots, such as those for ships, storage tanks, and tank lorries, and proposes an adaptive belt tension robot equipped with a 4-point supported belt mechanism capable of sanding variable curved surfaces. In addition, a sanding normal force prediction formula is proposed to describe the sanding performance of the contact surface. This equation consists of the concentrated load function due to the belt movement and the normal force due to the vertical and horizontal elongation of the belt. A video image analysis was performed to calculate the sanding area. Therefore, we determined whether the area was uniformly sanded. The dimensions of the test bench (W $\times$ D $\times$ H) were 1700 mm $\times$ 1450 mm $\times$ 900 mm. Experiments were performed using the proposed techniques on convex specimens with radii of 725, 1000, and 2100 mm. The sanding performance was improved by 43 $\%$ compared with that of a general belt-sanding robot.

## Introduction

**Figure 1:**
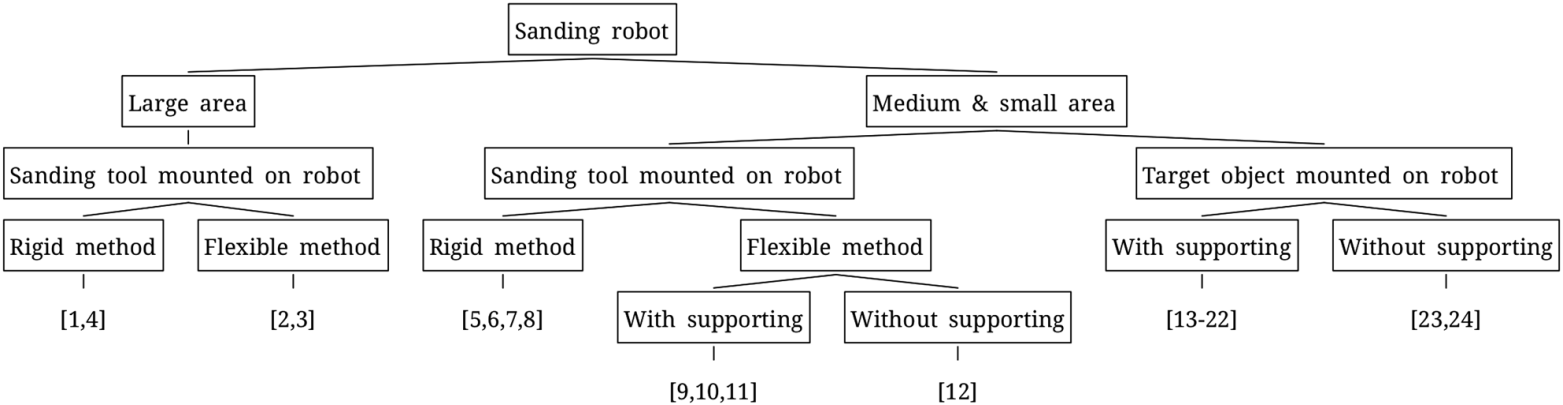
Classification tree for existing sanding robots.

Generally, when sanding an object over a large area, a sanding robot that moves as if drawing a line along a curved surface is used. In this case, a line that is as thick as possible (i.e., a large sanding area) is used to maximize the sanding area, and the trajectory of the sanding robot must be moved to overlap the previous polishing trajectory and ensure uniform sanding quality. Sanding robots can be classified into two types: those used when the object is larger than the robot, and those utilized when the object is smaller than the robot, as shown in Fig. 1. When the object is larger than the robot, it is inefficient to hold the object and sand it and thus, in most cases, robots are equipped with a sanding module. A robot that sands the welded part of a large pipe was developed using a rigid grinding wheel^1^. A large area of wood was sanded using a pad of sanding paper^2,3^. A rigid grinding head was used to sand the inside of a large pipe^4^. If the object is smaller than the robot, it can be sanded by mounting a sanding module on the robot or by mounting the object on the robot to reach the sanding location. If the robot is equipped with a sanding module, this can be a rigid module with a sanding head or grinder^5–8^ or a flexible module such as sanding paper or a sanding belt^9–12^. Currently, most flexible modules are supported by rigid driving wheels or plates attached to the back of a belt. In certain cases, flexibility is utilized to contact only the belt^12^. If the object is much smaller than the robot, it is mounted on the robot and contacted in the sanding area^13–22^. Currently, sanding belts are used in most cases. There is no difference from the rigid case when the supporting parts, such as the driving wheel behind the belt, are touched. In other cases, flexibility is utilized to contact only the belt^23,24^.

Most existing sanding robots focus on delicately sanding small objects. However, in industry, there are many sanding objects with large curved surfaces made of hard metals, such as iron. Examples include large ships, water tanks, and tank lorries. Robot sanding of such large curved metal surfaces cannot be performed with conventional methods. Mounting a large object on a robot is not possible. There have also been attempts to reduce errors by using a robot control that analyzes and interpolates surface inclination or profiles through 3D scanning^7,8^. However, the rigid sanding module is not suitable for sanding a curved object. To this end, a flexible sanding module should be used. However, sanding paper has too little deformation, and it is difficult to deform it according to the curvature. A sanding belt is not suitable for various curvatures because it is mostly in contact with the support part on the back. In addition, in terms of the size of the sanding module, the area of the sanding module is too small compared to the size of the object; therefore, the area that can be worked on at once is small. In this case, not only does the work efficiency decrease but there is also a gap between the sanded surfaces; therefore, the surface is not uniform. If the object is a soft tree, there is no significant difference; however, if a large object made of metal is the target, a grain will be formed on the sanded surface, resulting in uneven results. The performance of machining operations, such as milling or grinding, is generally evaluated using the material removal rate (MRR)^25,26^. This is an important indicator, but sanding cannot be evaluated only by MRR. Depending on the operation, the terms polishing, sanding, and grinding are combined and used. Polishing mainly refers to the final shining process, sanding refers to the peeling of rust or exterior materials, and grinding refers to grinding deeper into the surface. Because sanding does not deal with raw materials like other machining operations but is a method for finishing an object that has already been processed, it is more important to sand uniformly than sand abundantly. Belt sanding as a finishing process can achieve a high material removal rate with fine surface quality. In addition, it can be operated at a relatively low temperature, which benefits many intractable materials, such as aluminum and titanium alloys^27^. In cases where high material removal and low sanding temperatures are required, particularly in those with complex-shaped targets, belt sanding is the optimal choice^28^. The flexibility of the belt is advantageous for structures with complex shapes. Therefore, to sand large curved metal surfaces uniformly, we decided to use a belt contact method that minimized the support surface^12,23,24^. Unlike the existing method, the size of the sanding module is increased for large-area sanding. The optimal value was determined experimentally based on the Taguchi method. The Taguchi method is commonly used to optimize sanding surfaces^29–31^. This measurement method uses vision, not the MRR, to verify whether the sanding surface is uniform. Because the color or contrast of a surface varies before and after sanding, the results are often analyzed using vision in sanding experiments^7,8,32–34^.

When sanding physically, most methods operate by rotating a sanding pad, sanding stone, or sanding belt using a motor. Nonetheless, uniform sanding is difficult because the magnitude of the friction force varies depending on the rotation direction of the motor or the amount and direction of contact between the target and the sanding surface. If the size of the target is small, it is possible to sand the entire target surface simultaneously; therefore, this is not a significant problem. However, if the size of the object increases, the sanding state is affected because the object receives a different force at each position of the abrasive. It is necessary to correct the impact of the rotation of these motors during sanding so that the target objects are uniformly sanded.

**Figure 2:**
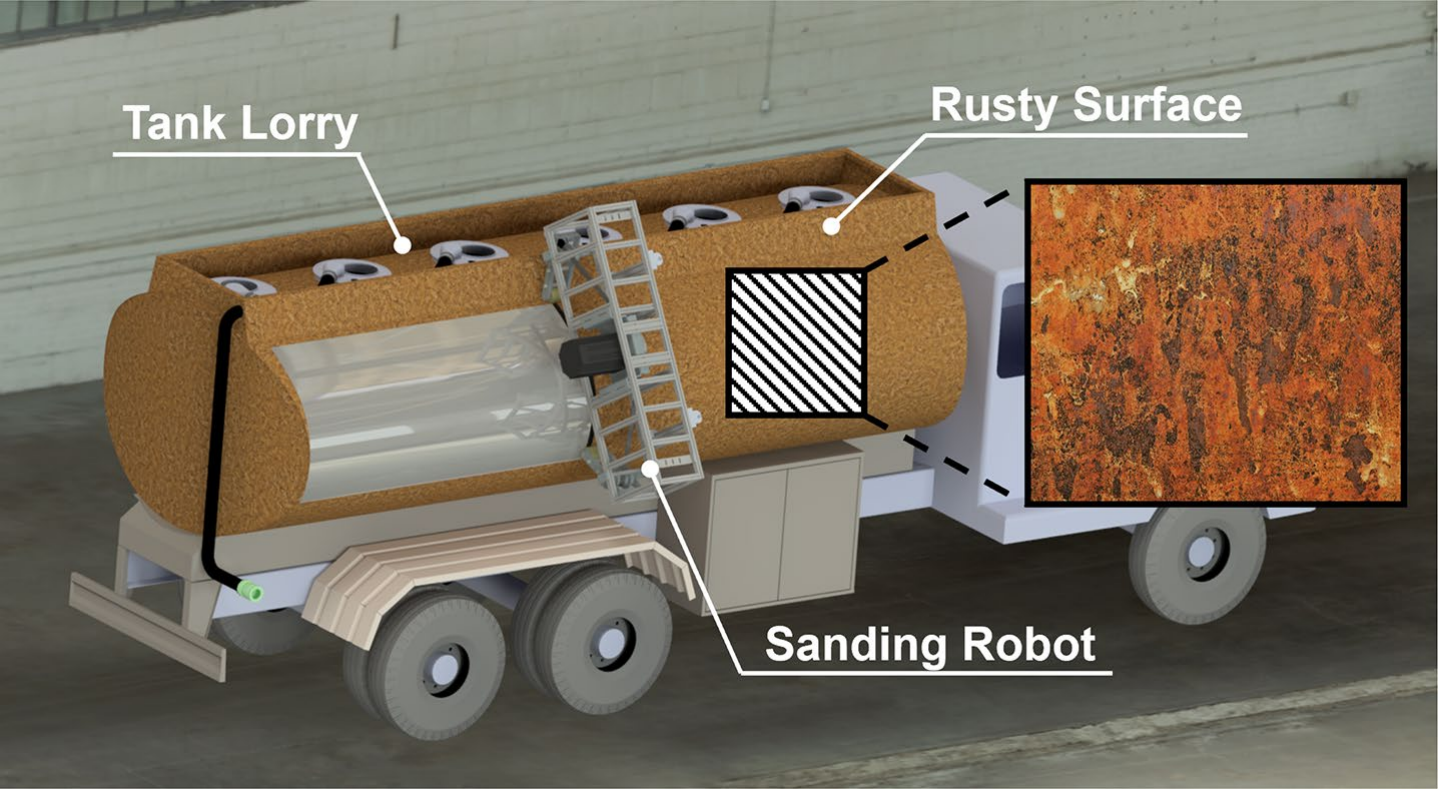
Concept design of a belt sanding robot.

Therefore, a 4-point supported belt sanding robot that uses the flexibility of the sanding belt was proposed to reach surfaces with various curvatures while performing large-convex area sanding. The 4-point supported belt sanding robot overcomes the limitations of sanding robots moving in point contact with a sanding object and enables rapid and uniform sanding, as shown in Fig. 2.

To sand a large curved surface uniformly, the force generated by the system must be uniform because sanding is performed in proportion to the force applied to the curved surface of the target. Although an accurate prediction is essential for the development of variable large-area sanding robots, few studies have been conducted on the distribution of the sanding force acting on the position of a variable curved surface and the imbalance of the sanding force^35–37^. A study was conducted on tension distribution in existing belt systems^24^. However, in the tension calculation, it was assumed that the contact angle was close to zero because the size of the sanded object was small. The tension of the belt was calculated in a situation where the belt was floating without an external influence because it did not contact the pulley, idler, or curved surface. These existing analyses are not suitable for large-area sanding. For large-area sanding, the robot requires verifying the distribution of the belt force in contact with a curved surface.

Therefore, a 4-point supported belt sanding robot with the sanding belt is proposed to reach surfaces with various curvatures while performing large-convex area sanding. The 4-point supported belt sanding robot overcomes the limitations of sanding robots moving in point contact with a sanding object and enables rapid and uniform sanding. In addition, a sanding normal force prediction formula is proposed to describe the sanding performance of the contact surface, and an image analysis technique capable of quantitatively measuring the sanding area is proposed. This paper consists of six sections. “Development of belt sanding kinematics” Section describes the mathematical model of belt sanding. “Experimental setup” Section includes the experiment setup. “Optimal design method” Section shows the optimization method for the sanding robot. “Experiment results and discussion” Section is the result of the experiment and discussion. Finally, “Conclusion” Section is the conclusion of the paper.

## Development of belt sanding kinematics

**Figure 3:**
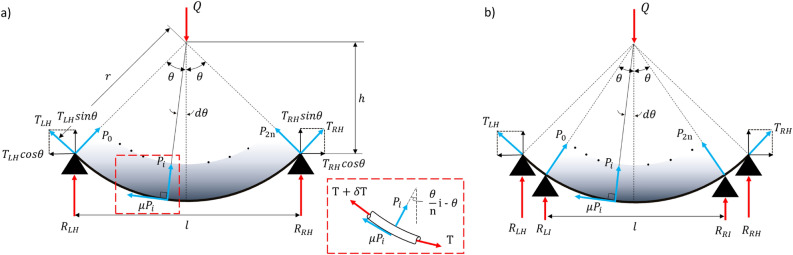
Force equilibrium of sanding belt. (**a**) 2-point support belt sanding condition. Rex box is the free-body diagram of the micro-scale belt. (**b**) 4-point support belt sanding condition.

For uniform sanding, it is important to determine the contact surface pressure acting on a large-area curve (i.e., the normal force acting on the belt). Two main factors should be considered regarding the surface pressure acting on the belt. The force equilibrium of the 2-point support belt sanding system is shown in Fig. 3a. It shows the force balance that occurs when the belt comes into contact with a bent object. The main forces in belt sanding are the force balance of the axial force moved during sanding (i.e., the relationship between the applied total load *Q* and the reaction forces $R_{LH}$ and $R_{RH}$) and the normal force balance of the belt for large-area uniform sanding (i.e., the relationship between the belt tension *T*, friction force $F_{\mu }$, and normal force *P*).

As the contact area with the curved object increases (i.e., the contact angle of the belt increases), the belt tension also increases. The belt bent according to the curvature in contact with the curved surface was divided into 2n equal parts, and the micro-unit belt was expressed in the red box of Fig. 3. At the free body diagram on the $i^{th}$ belt part, $\delta T$ can be assumed as 0 because the value is very small to 0.

The belt tension *T* is proportional to the stiffness *k* of the belt and the total elongation $\delta _l$ in the longitudinal direction. *A* is a system constant. The relationship of T can be described as Eq. (1).

1 $$\begin{aligned} T = A\cdot k \cdot {\delta _l} = A\cdot k \cdot (2r\theta - 2r \sin \theta ) \end{aligned}$$

The $i^{th}$ belt part’s normal force $P_i$ can be expressed as

2 $$\begin{aligned} P_i=T \cdot sin({d \theta }) = A\cdot k \cdot (2r\theta - 2r \sin \theta )\sin \left( {\frac{\theta }{n}i - \theta }\right) \end{aligned}$$

It was assumed that the belt was fixed at the support points at both ends and was tensioned only in the vertical direction shown as black triangles in Fig. 3. The amount of deformation of the belt and the tension of the belt are proportional. However, the vertical force that the belt receives is different when it is tensioned only horizontally by a horizontal external force and when it touches the curved surface and is tensioned by a vertical distribution load. Due to this relationship, the vertical force of the belt was insufficient to be expressed in the equation of only the amount of deformation, so the vertical component was considered assuming that the support points of both ends were fixed and tensioned only in the vertical direction. The more the belt is tensioned, the larger the received force, so in Eq. (2), *k* can be assumed to be proportional to the vertical displacement. If *B* is system constant, *k* is expressed as follows.

3 $$\begin{aligned} k = B\left( r cos\left( {\frac{\theta }{n}i - \theta }\right) - h\right) \end{aligned}$$

Because the normal force varies with the lengthwise elongation of the belt, the same frictional force does not act at the contact area between the belt and the object. In addition, because the belt can be regarded as an elastic body with a certain belt modulus *k*, the normal force increases proportionally with the amount of deformation in the normal direction of the belt. The relationship between the frictional force of the belt $F_{\mu }$ and the normal force *P* is as shown in Eq. (4).

4 $$\begin{aligned} F_{\mu } = \mu \left( r cos\left( {\frac{\theta }{n}i - \theta }\right) - h\right) \propto P \end{aligned}$$

Substituting Eq. (3) in Eq. (2), $P_i$ is expressed as follows.

5 $$\begin{aligned} P_i= 2AB\left( r cos\left( {\frac{\theta }{n}i - \theta }\right) - h\right) (2r\theta - 2r \sin \theta )\sin \left( {\frac{\theta }{n}i - \theta }\right) \end{aligned}$$

**Figure 4:**
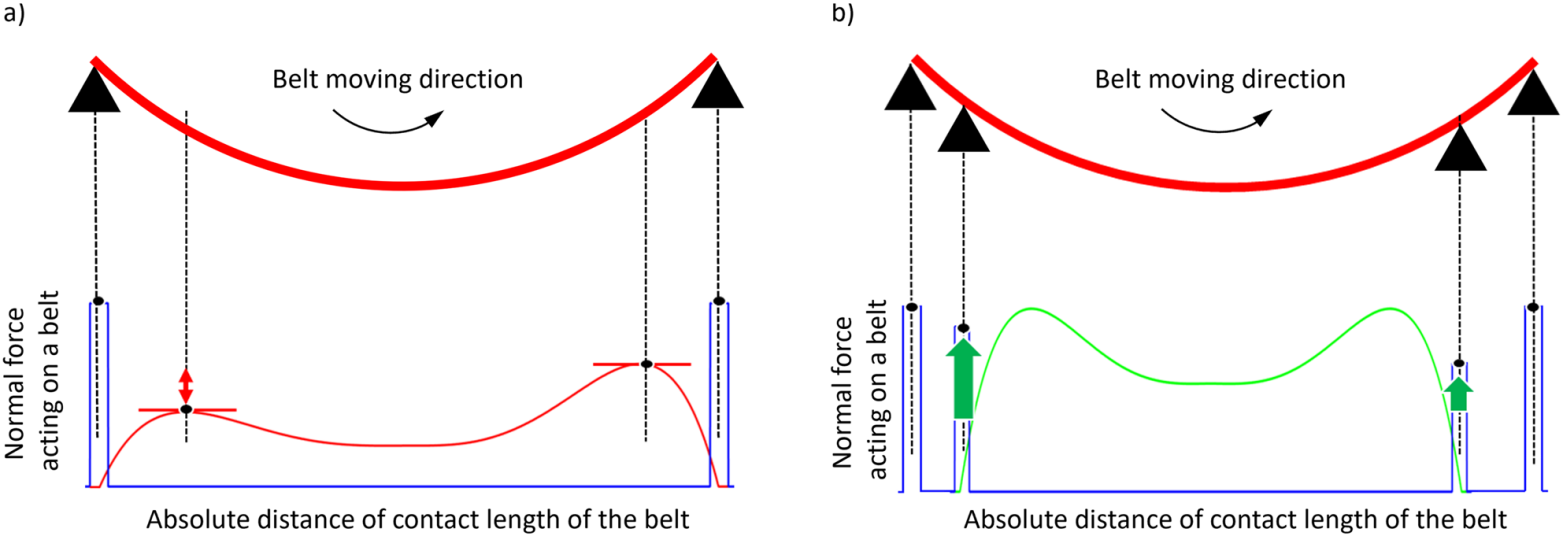
Estimated curve for sanding normal force. (**a**) Non-uniform distribution, (**b**) Desired normal force distribution.

This Eq. (5) expresses the belt normal force between the two support points. At the support points at both ends, the belt is affected by the reaction force, and the total belt’s normal force increases in proportion to the magnitude of the reaction force. Ignoring the deviation of the left and right tensions caused by rotation, the equilibrium of the vertical force at the support point for the belt movement by mechanical operation was considered, as shown in Eq. (6).

6 $$\begin{aligned} Q=R_{LH}+R_{RH}+R_{LI}+R_{LI}+2T\sin {\theta } \end{aligned}$$

where $Q, R_{LH}, R_{RH}, R_{LI}, R_{RI}, T$ denote the applied load, reaction force on the left-hand side, reaction force on the right-hand side, desired input reaction force on the left-hand side, desired input reaction force on the right-hand side, and tension, respectively. The estimation of the normal force acting on the belt-sanding robot using Eqs. (5) and (6) is shown in Fig. 4. In the case of 2-point support sanding shown in Fig. 3a, $R_{LI}$ and $R_{RI}$ do not exist. Therefore, the result of Fig. 3a is shown in Fig. 4a. The result of Fig. 3b is shown in Fig. 4b.

A high normal force, which is the blue line in Fig. 4a,b, is generated at the left and right ends that contact the object by moving the belt mechanism; however, it can be predicted that a severe nonuniform normal force will occur inside the belt contact area, as shown in the red line in Fig. 4a. The purpose of this study is to create a uniform sanding normal force, as shown in the green line in Fig. 4b by constructing a mechanism that gives a uniform sanding force to a large-area object with variable curvature. The reaction force sizes of the two support points are different. In the blue line in Fig. 4b, the right and left second peak forces will be generated owing to the additional supporting arms. This creates a more uniform form of force overall. To create a uniform sanding normal force, a 4-point supported belt sanding robot with two additional auxiliary support points was developed.

## Experimental setup

This section describes the settings and parameter selection used in the experiments.

### Design of 4-point supported belt sanding robot

**Figure 5:**
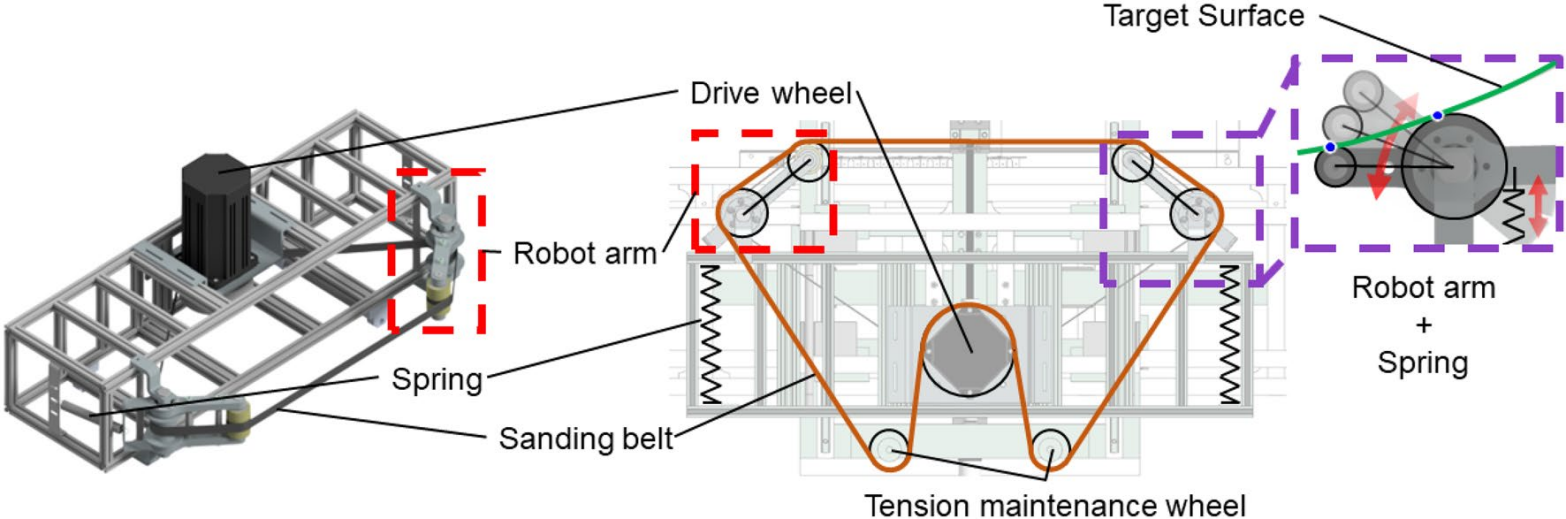
Schematic of the 4-point supported belt sanding robot.

A schematic of the 4-point supported belt sanding robot proposed for curved large-area sanding is shown in Fig. 5. The drive wheel motor rotates the sanding belt clockwise. The specifications of the drive wheel motor (S13-D2021-4P-WG-Z, Motorbank) are 2 kW and 2000 rpm. The robot arm consists of two contact wheels. The contact wheel at the end of the robot arm is adjusted to the curvature and the tension is maintained. It comes into contact with the target surface at the contact point of the robot arm. The contact point is described as the blue dot in Fig. 5 purple box, which is the same as the four support points in Fig. 4b.

The robot arm uses a spring structure to maintain the reaction force upon contact under the user’s conditions. The length of the belt that shrinks as the robot arm folds are compensated by a tension maintenance wheel fixed to the robot body. The tension maintenance wheel is fixed to maintain the belt tension when the moving stage moves forward. Without the tension maintenance wheel, the belt would deviate as its length changes when the arm is folded.

### Test bench design

**Figure 6:**
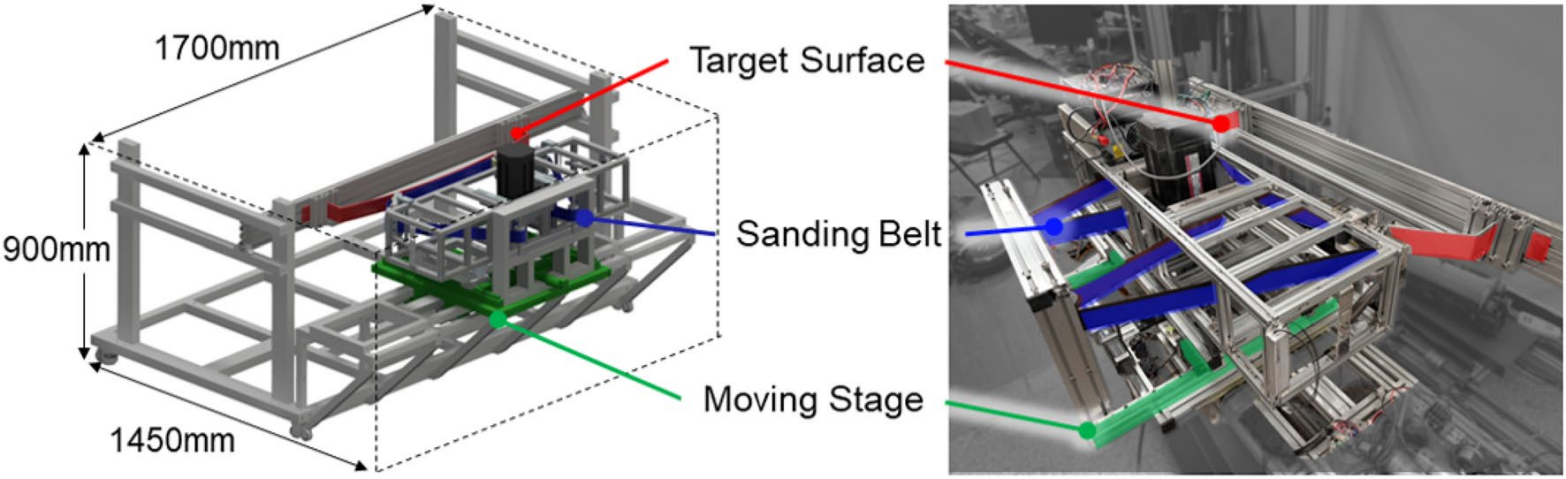
Sanding test bench design.

**Figure 7:**
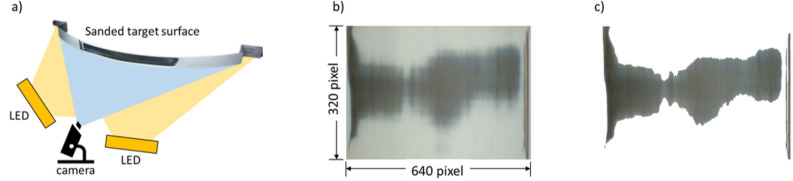
The method of video image analysis to calculate the sanded area. (**a**) Visual inspection bench design, (**b**) Resized image example, (**c**) Blacked pixel example.

- Sanding test setup
  The curvature of the target curved surface adheres to the European Union’s ADR Agreement for the International Carriage of Dangerous Goods by Road, with a long axis diameter of at least 1.5 times the short axis diameter^38^. Based on this requirement, three suitable curvatures were selected. The curved samples were made of 5.0 mm thick steel as shown in the red lined target surface in Fig. 6. The target curved surface was constructed from steel with a thickness of 5t. An aluminum profile frame was built around the target curved surface container to accommodate the moving stage, boards, and motors. Various parts for securing the motor, roller, and contact wheel were 3D printed using a Stratasys 170 printer, utilizing ASA filament as the printing material. The sanding belt utilized an aluminum oxide sanding belt with a width of 50 mm and a grit size of 100. The motor driving the sanding belt was a BLDC motor (TM13-D2021-S-4P-K-SG DC24V 2KW, motorbank), controlled by an MD2K motor driver. The power supply ensured stable power delivery. The moving stage allowed for movement of up to 200 mm and enabled the robot to approach the curved surface. A constant force of 20 N was applied to the moving stage against the wall, ensuring consistent contact between the robot and the target curved surface and thus facilitating the sanding process. The test bench used in this study is shown in Fig. 6. When experimenting with 2-point support sanding, the experiment was conducted by removing one contact wheel of the robot arm and setting the 2 points in contact with the curved surface.
- Calculation of sanding area through video image analysis
  An imaging technique was applied as a method for calculating the sanding area after sanding, as shown in Fig. 7. The experiment was repeated three times under the same measurement conditions. In the workpiece image at the same location, a specific area was resized to the same size (640 x 320 pixels) using the resize process. The image was further categorized into H (Hue), S (Saturation), and V (Value), distinguishing between sanded and unsanded surfaces. The number of black pixels representing the sanded area was divided by the total number of pixels in the image (number of pixels of resized area: 290133). A percentage value representing the extent of sanding was obtained, as expressed in Eq. (7).
  7 $$\begin{aligned} Sanded \; area(\%) = \frac{Black \; pixels}{Resize \; total \; pixels}\cdot 100 \end{aligned}$$

## Optimal design method

The design parameters and user conditions for adopting the Taguchi method were set. Subsequently, a suitable orthogonal array was selected, and a combination of design parameters for testing was determined.

### Setting design parameters and user conditions

**Figure 8:**
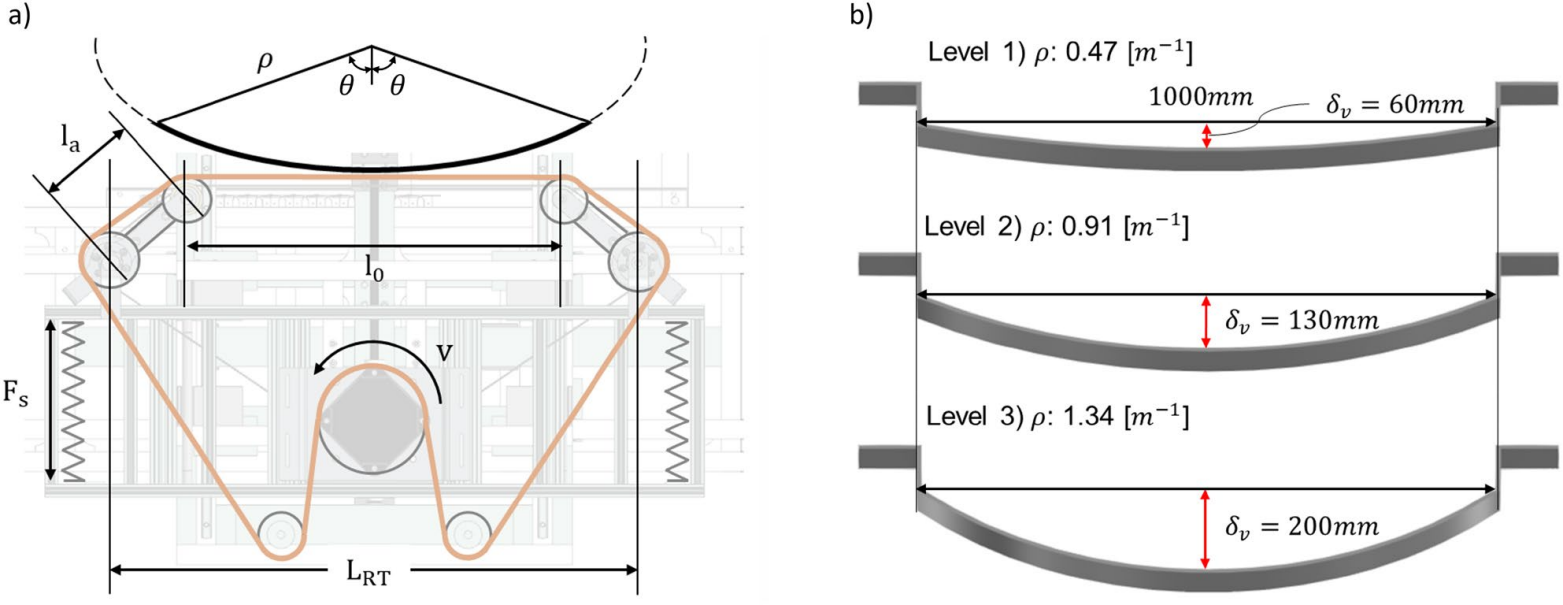
The notation of variables for optimization. (**a**) Design parameter. (**b**) User condition: the curvature of the target convex surface.

The belt system supporting force $F_s$, the speed of the belt rotation motor *V*, and the distance between the major belt travel point $L_{RT}$ were selected as the main operational parameters of the sanding robot. $l_a$ is the robot arm length and $l_0$ is the robot length that contacts with the target surface. The spring force $F_s$ was adopted as the design variable based on theoretical calculations. These design parameters are shown in Fig. 8a. For the user condition, a curvature level of 3 was set. Three target samples with widths of 60 mm were prepared. The curvature $\rho$ of the samples were 0.47, 0.91, and 1.34 $m^{-1}$, which represent curved products with large diameters of approximately 1500, 2200, and 4200 mm as shown in Fig. 8b.

It was determined that adding a force in the direction in which the target convex surface was pushed with the robot arm was advantageous for large-area sanding. Springs were used to generate forces in this direction; therefore, spring forces were selected as the design variables. The motor speed *V* and the overall robot length $L_{RT}$ generally positively impact the sanding performance as their values increase. However, when the objective function aims to maximize the sanded area in a uniform state, it is uncertain whether the objective function will increase proportionally with these values. When sanding the target with a belt, the quality of the surface is influenced by factors such as the centrifugal force, which causes a slight belt deviation from the pulley at higher speeds, thereby affecting the belt tension. Moreover, an increased speed can intensify disturbances and vibrations in the robot itself, which can be detrimental to achieving uniform sanding. While a longer robot length allows for a larger area to be sanded simultaneously, it also means more belt contact with the target curved surface without a support section, potentially compromising tension maintenance and uniform sanding. Therefore, careful consideration is required when adjusting these parameters.

The belt elongation according to the curvature is shown in Table 1. The 4-point supported belt sanding robot aims to enhance the sanding performance by adapting to various curvatures. Because specific curvatures are not specified in the actual usage environment, the curvature was selected as the user condition. The curvature of the tank lorries was applied based on the criteria stated in the European Union’s ADR Agreement for the International Carriage of Dangerous Goods by Road, which stipulates that the diameter of the long axis of a tank lorry should be at least 1.5 times the short-axis diameter^38^. The value of design parameters is shown in Table 2. It is worth noting that the interval between spring forces may not be an integer value as it was adjusted experimentally based on the distance of spring extension.

**Table 1:** Belt elongation and angle by target surface curvature.

		Level 1	Level 2	Level 3
$\delta _H$	Longitudinal belt elongation (mm)	9.4	38.1	95.8
$\delta _V$	Vertical belt elongation (mm)	59.6	120.3	192.3
$\theta$	Central angle (^∘^)	27.2	27.1	42.1

**Table 2:** Design parameters and user conditions for 1st experiment.

	Design Parameter	Level 1	Level 2	Level 3
$F_s$	Spring force (N)	56	80	104
*V*	Motor speed (rpm)	250	350	450
$L_{RT}$	Robot total length (mm)	780	760	740
	User Condition	Level 1	Level 2	Level 3
$\rho$	Target Curvature ($m^{-1}$)	0.47	0.91	1.34

### Selection of orthogonal array

In the Taguchi method, an orthogonal array is used to combine the design parameters. This approach allows the determination of the optimal combination of design parameters with fewer experiments, as it is not necessary to test all possible parameter combinations. Different types of orthogonal array are available, such as ${\textbf{L}}_4$($2^3$), ${\textbf{L}}_8$($2^7$), ${\textbf{L}}_12$($2^11$), and ${\textbf{L}}_16$($4^1$)^39^. In this experiment, because there were three levels for each of the three design parameters, an ${\textbf{L}}_9$($3^4$) orthogonal array was selected. Each curvature level was tested using different parameter combinations to maximize the sanded area, which was the objective function. The experiment was conducted three times to ensure repeatability of the results.

The experimental results were analyzed using the signal-to-noise (S/N) ratio to determine the optimal design parameters. Three methods can be used to analyze the S/N ratio: “the-nominal-the-better,” “the-larger-the-better,” and “the-lower-the-better.” In this experiment, because a larger sanded area is desirable, the “the-larger-the-better” method was used. The S/N ratio was calculated using Eq. (8).

8 $$\begin{aligned} S/N \; ratio = -10 \cdot log\left| \frac{\left( \frac{1}{y_1}\right) ^2+\left( \frac{1}{y_2}\right) ^2+ \cdot \cdot \cdot +\left( \frac{1}{y_n}\right) ^2}{n}\right| (dB) \end{aligned}$$

where $y_1$ and $y_2$ $\cdot \cdot \cdot$ $y_n$ represent the results of the experiments (i.e., the sanded area measured by the vision camera), and *n* is the number of experiments conducted for each design parameter combination.

## Experiment results and discussion

A comparison of the sanded areas as a result of the 1st sanding experiment is presented in Table 3. Table 4 lists the design parameter combinations and S/N ratios obtained using the ${\textbf{L}}_9$($3^4$) orthogonal array of the Taguchi method. The sanded area was calculated using vision detection, and each experiment was conducted for 3 minutes.

**Table 3:** Results of the 1st experiment—comparison of sanding area according to design variables.

Experiment number	$F_s$	*V*	$L_{RT}$	Sanded area (%)		
				$$\rho $$		
	Level			Level 1	Level 2	Level 3
1	1	1	1	6.65	22.62	38.33
				6.61	22.63	39.62
				6.49	22.24	38.89
2	1	2	2	20.6	23.86	35.91
				20.47	23.88	37.04
				20.19	23.4	36.46
3	1	3	3	12.76	25.37	38.57
				12.69	25.39	39.81
				12.48	24.86	39.01
4	2	1	2	17.27	27.37	36.34
				17.24	27.36	37.54
				16.97	26.93	36.77
5	2	2	3	17.77	25.46	37.92
				17.73	25.45	39.13
				17.39	24.95	38.47
6	2	3	1	16.9	33.72	41.39
				16.89	33.73	42.72
				16.58	33.17	41.92
7	3	1	3	14.44	23.02	42.33
				14.45	23.03	43.7
				14.2	22.57	42.75
8	3	2	1	23.24	25.72	41.14
				23.24	25.74	42.52
				22.8	25.07	41.73
9	3	3	2	21.81	29.99	43.04
				21.88	29.99	44.55
				21.44	29.31	43.55

**Table 4:** Comparison of signal-to-noise (S/N) ratio by design parameter level of the 1st experiment.

Design parameter level	$F_{s1}$	$F_{s2}$	$F_{s3}$	$V_1$	$V_2$	$V_3$	$L_{RT1}$	$L_{RT2}$	$L_{RT3}$
S/N ratio (dB)	23.62	27.52	27.76	23.69	27.97	27.17	24.14	28.02	26.27
deviation	4.14			4.28			3.88

**Figure 9:**
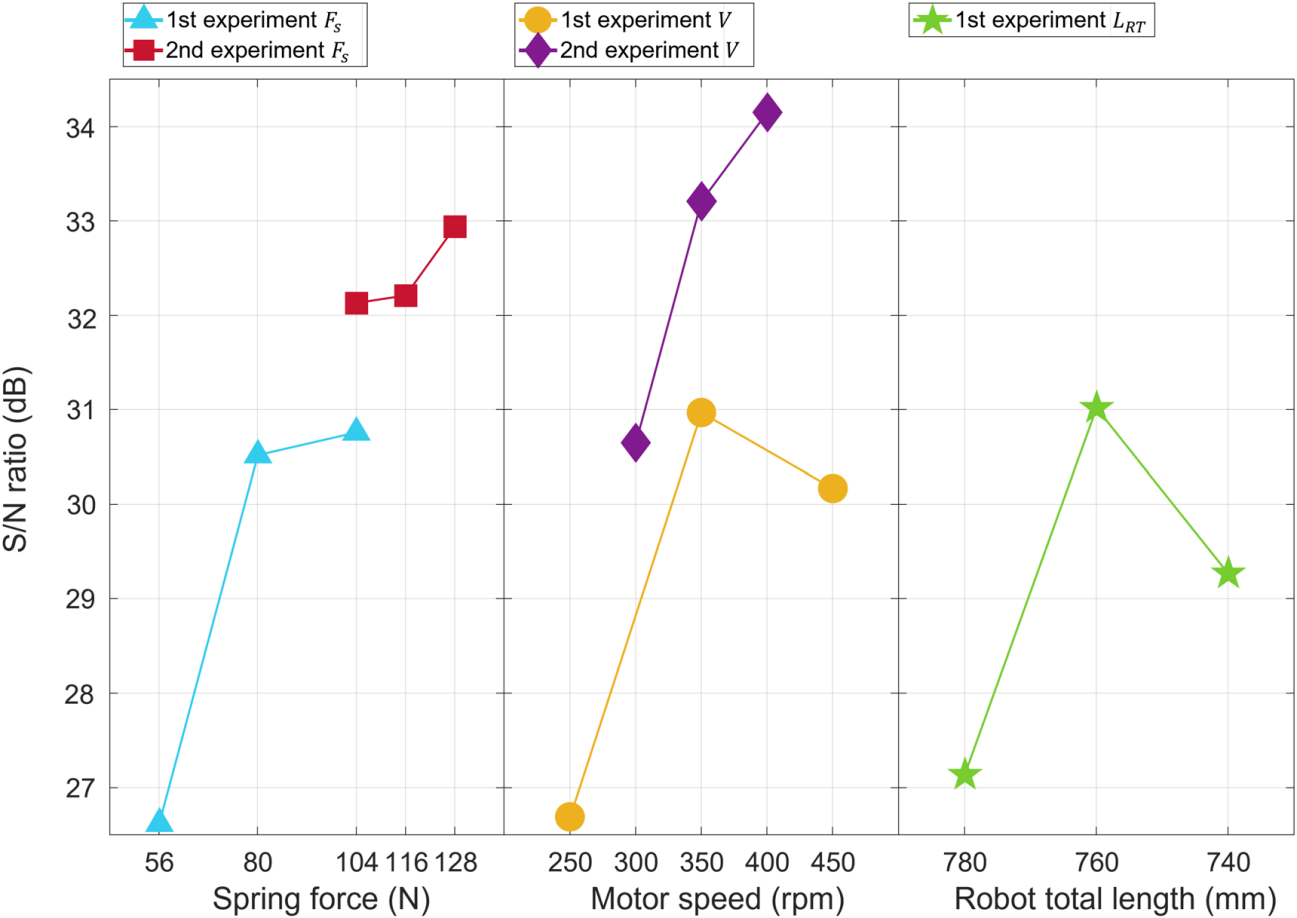
S/N ratio of ${\textbf{L}}_9$($3^4$) Taguchi orthogonal array.

### Signal-to-noise ratio analysis

The S/N ratio indicates the sensitivity of a parameter. The large S/N ratio in the robust optimal design indicates that the variation in the design variable by user condition (i.e., noise) is small, and simultaneously, the design variable itself implements the characteristics (i.e., signals) well. This implies that the higher the S/N ratio of a parameter, the greater its influence on the experimental results. The blue lines corresponding to the 1st experiment shown in Fig. 9 are visual representations of the S/N ratios in Table 4. The spring force was the most sensitive parameter. For the spring force, as the value of the design variable increased, the S/N ratio tended to increase. In terms of the motor speed and robot total length, the S/N ratio showed a maximum value at level 2. Through the 1st experiment, the optimal values of the motor speed and robot total length were found, and it was determined that further exploration should be performed to determine the optimal value for the spring force.

**Table 5:** Design parameters and user conditions for the 2nd experiment.

	Design Parameter	Level 1	Level 2	Level 3
$F_s$	Spring force (N)	104	116	128
*V*	Motor speed (rpm)	300	350	400
$L_{RT}$	Robot total length (mm)	760		
	User Condition	Level 1	Level 2	Level 3
$\rho$	Target Curvature ($m^{-1}$)	0.47	0.91	1.34

The spring force was adjusted from level 3 in the 1st experiment to level 1 in the 2nd experiment, and the experiment was conducted at a higher spring force. The motor speed determined that level 2 was the optimal value in the 1st experiment, but a better optimal value could be obtained from nearby values. Therefore, in the 2nd experiment, the experiment was conducted at narrower intervals based on the level 2 value in the 1st experiment. In the case of the robot’s total length, the experiment was conducted by adjusting the position of the robot arm at the total robot length of 1000 *mm*. However, the gap between the levels is 20 *mm*, which is quite small. The deviation of the S/N ratio is relatively small shown as in Table 4. That means the sensitivity of the S/N ratio is less than that of the other design variables. Therefore, rather than conducting the 2nd experiment in more detail, the results of the 1st experiment were confirmed as the optimal value, and the 2nd experiment was conducted by fixing the value.

**Table 6:** Results of the 2nd experiment—comparison of the sanding area according to design variables.

Experiment number	$F_s$	*V*	$L_{RT}$	Sanded area (%)		
				$$\rho $$		
	Level			Level 1	Level 2	Level 3
1	1	1	2	16.24	41.46	57.26
2	1	2	2	23.89	27.35	51.63
3	1	3	2	22.93	42.19	49.91
4	2	1	2	18.37	20.98	38.62
5	2	2	2	23.29	41.75	49.25
6	2	3	2	29.86	46.59	45.93
7	3	1	2	15.99	41.94	51.18
8	3	2	2	32.92	35.75	41.47
9	3	3	2	34.08	35.26	56.33

**Table 7:** Comparison of S/N ratio by design parameter level of the 2nd experiment.

Design parameter level	$F_{s1}$	$F_{s2}$	$F_{s3}$	$V_1$	$V_2$	$V_3$
S/N ratio (dB)	29.13	29.21	29.94	27.65	30.21	31.15
deviation	0.81			3.5		

Based on this, the design variables for the 2nd experiment were set as listed in Table 5. A comparison of the sanded areas resulting from the 2nd sanding experiment is presented in Table 6. Visual representations of the S/N ratios in Table 7 are shown in Fig. 9. In the 2nd experiment, the slope of the spring force decreased, but the S/N ratio value also increased as the level increased. Experiments were conducted at a higher level to determine the optimal spring force. However, in the 2nd experiment, if a spring force of 3 or higher was applied, the force acting on the target curved surface of the robot arm became so strong that the belt was strongly pressed at one point. In this case, the sanding belt became fixed with a strong force and did not rotate, resulting in no sanding. This was additionally verified at smaller intervals; however, in the 2nd experiment, the sanding belt did not rotate if it was slightly higher than the spring force. Therefore, for the spring force, level 3 of the 2nd experiment was selected as the optimal value, and sanding could not be performed at a larger value. The motor speed showed the highest value at level 3 in the 2nd experiment, and this was selected as the optimal value. The optimal design variables were a spring force of 127.52 N, a motor speed of 400 rpm, and a total robot length of 760 mm.

### Comparison of theoretical analysis and experimental results

**Figure 10:**
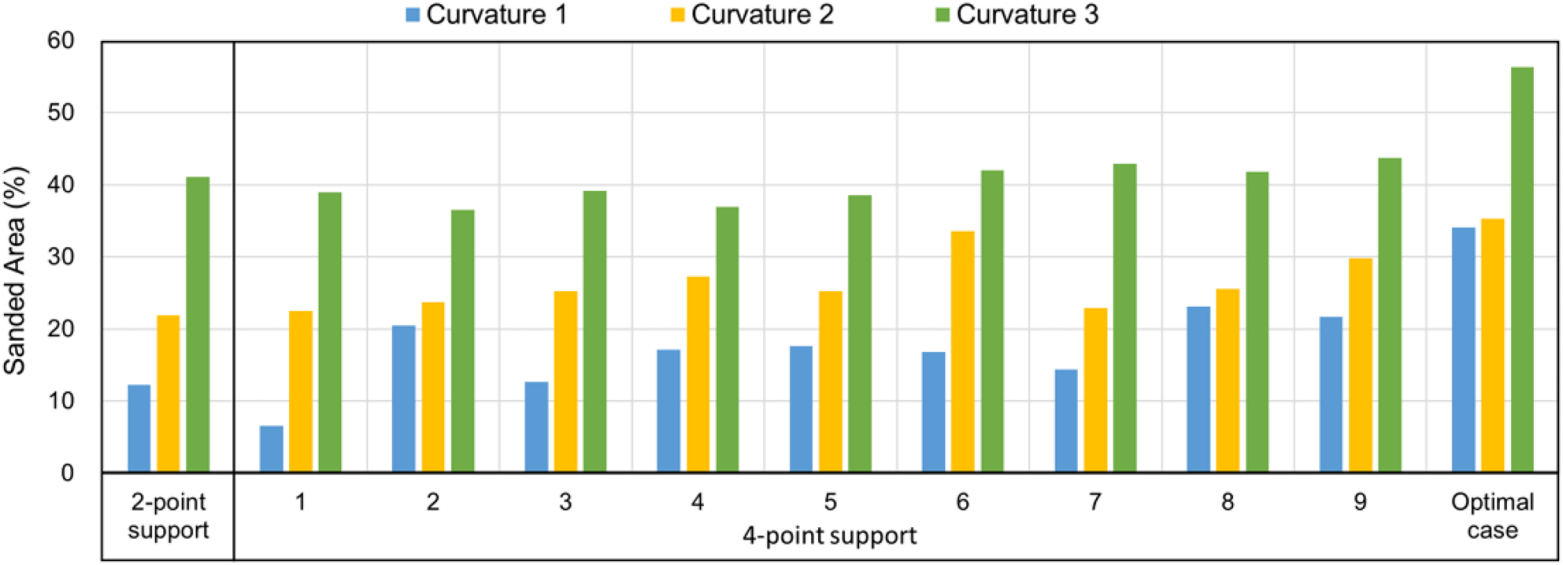
Comparison of sanding area by test conditions.

The optimal values of the design variables were experimentally obtained. By applying this, the sanding area was confirmed when sanding was carried out using a 2-point and a 4-point supported belt sanding robot, and the optimal design variables were applied. The sanding area of the 2-point and 4-point support belt sanding robot is shown in Fig. 10. 1 through 9 are the results of the sanding area of the 1st experiment, and the optimal case is the area per sand when optimized design variables are applied. The measured abrasive area was 34.08$\%$ at curvature 1. It was measured at 35.26 $\%$ at curvature 2 and 56.33 $\%$ at curvature 3. As a result of comparing the sanded area of the traditional 2-point support mechanism and that of the 4-point support mechanism, it was confirmed that the sanding area was improved by 43 $\%$ in the 4-point support mechanism.

**Figure 11:**
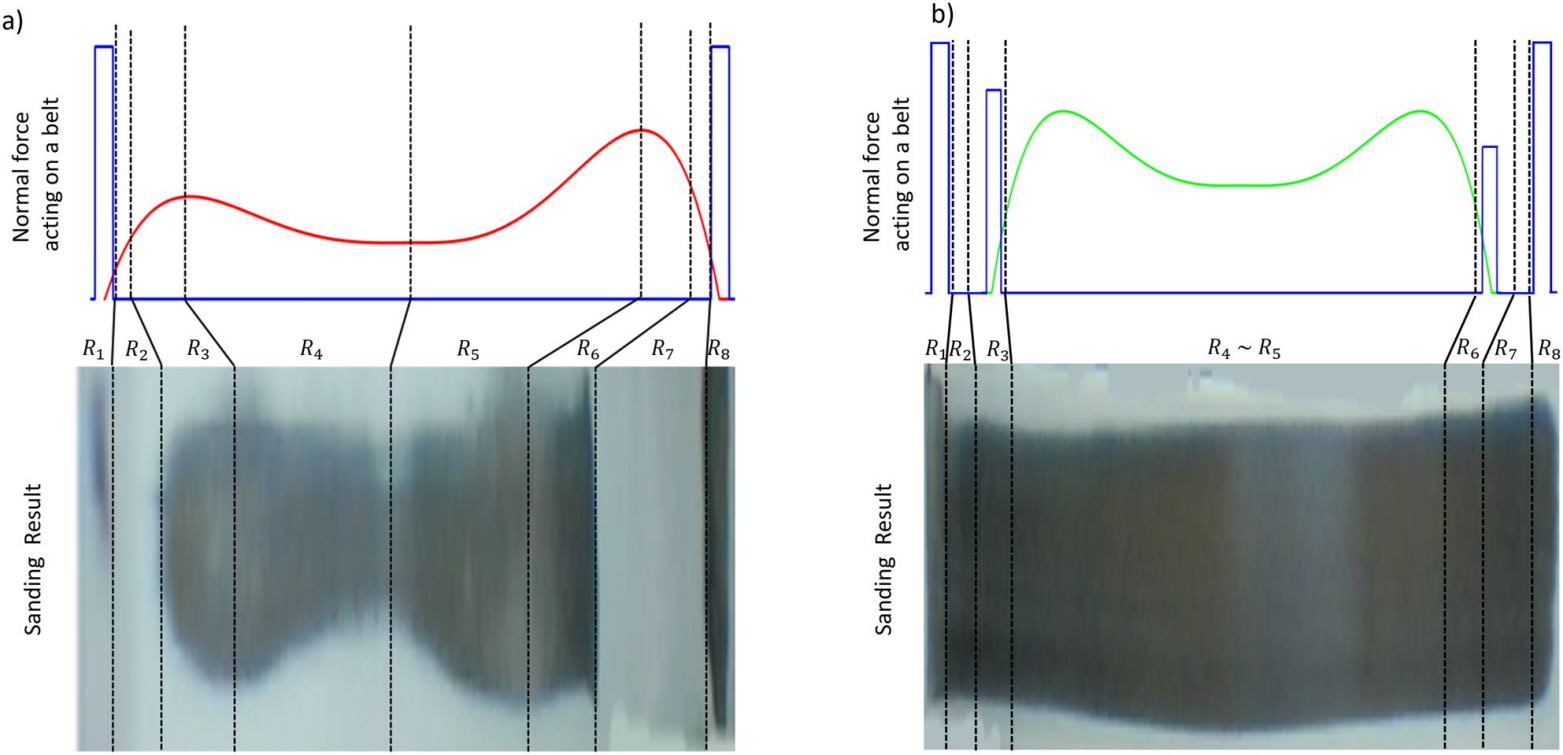
Comparison between the theoretical force distribution curve (top) and the actual sanding image (bottom) at curvature 0.91. (**a**) sanding result using the 2-point supported belt sanding robot, (**b**) sanding result using the 4-point supported belt sanding robot.

This was compared with the force acting on the theoretically obtained sanding area. Fig. 11a shows the case of sanding using the 2-point support. The graph of the normal force acting on the theoretically calculated belt and the image of the curved surface after sanding obtained from the experiment were compared. They exhibited a similar form; thus, it was confirmed that the theoretical calculation was valid. This was compared by dividing it into eight regions from the left, from $R_1$ to $R_8$. In both end regions, that is, regions $R_1$ and $R_8$, a strong normal force was generated under the influence of the reaction force. Therefore, it can be observed from the experimental image that the sanding was strong. The difference between the theoretical calculations and experimental results is in regions $R_2$ and $R_7$. In the experiment, no sanding was observed in these regions. Therefore, in the theoretical calculations, the belt was viewed as a completely flexible body. However, an actual sanding belt is flexible because it has a hard side, such as sandpaper; however, it is difficult to see it as a very soft flexible body. For this reason, if two points are supported, they are strongly attached to the target curved surface in regions $R_1$ and $R_8$; however, unlike theory, they exhibit rigid characteristics in regions $R_2$ and $R_7$, so they float without touching the curved surface. Next, in regions $R_3$ and $R_6$, as in the calculation, the magnitude of the normal force gradually increases owing to the influence of tension. In the experimental image, the maximum normal force was shown in regions $R_3$ and $R_4$ and regions $R_5$ and $R_6$; thus, the sanding became relatively strong. In regions $R_4$ and $R_5$, the horizontal force becomes larger than the vertical tension at the intermediate point of the curved surface, and the normal force becomes smaller. The experimental results also showed that the sanding area near the center was relatively small. Thus, the reliability of the theoretical calculation results was confirmed.

The resulting image of the optimized values for the 4-point support robot is shown in Fig. 11b. Through one total of four support points at each end, the overall sanding area was found to be uniform and wider than that in Fig. 11a. Thus, it was confirmed that the 4-point support robot had an effect. Unlike the theoretical normal-force graph, we obtained a more uniform shape. This is because the theoretical normal force graph simply overlaps the normal force graph by the reaction force and the normal force graph by the tension by position.

The reaction force at the 4-point support point, represented by the blue line shown in Fig. 11b, is also expressed as a point. However, the force will affect the vicinity and appear in a linear form. In the actual experiment, the force acted as a combined force and acted more evenly. As a result of the experiment, the sanding area was more uniform and wider based on the results of the target convex curved surface.

## Conclusion

In general, sanding robots that move as if drawing a line along a curved surface are mainly used when sanding an object having a large area, but they require a long working time and it is difficult to secure a uniform sanding area. This study proposes an adaptive belt tension robot equipped with a 4-point supported belt sanding mechanism capable of variable curved surface sanding. In addition, we propose an equation for predicting the sanding normal force, which can explain the sanding performance of the contact surface. This equation consists of the concentrated load function due to the belt movement and the normal force due to the vertical and horizontal elongation of the belt. For calculating the sanding area, a video image analysis was introduced. Through this, it was possible to determine whether the sanding area was uniformly sanded. The dimensions of the test bench (W$\times$D$\times$H) were 1700 mm $\times$ 1450 mm $\times$ 900 mm. The sanding experiments were performed using the proposed techniques on convex specimens with radius of 725, 1000, and 2100 mm, and satisfactory results were obtained. We proposed a 4-point support sanding robot with a main 2-point support for belt movement and an auxiliary 2-point support for belt tension control.

- The experiment was successfully conducted on very large convex surfaces with diameters of approximately 1500, 2200, and 4200 mm.
- A sanding area calculation method using image analysis that can quantitatively evaluate the sanding performance was devised, and the S/N ratio for the test conditions and image area was used as a performance index of the sanding robot.
- The Taguchi method was applied with the three design variables (the belt supporting spring force, motor speed, and span of the robot’s arm were selected as the main operational parameters of the sanding robot), and the optimal conditions for the maximum sanding area were found.
- The optimal design parameters obtained experimentally were spring force 127.52 N, motor speed 400 rpm, and robot total length 760 mm. The S/N ratio for these optimal design parameters was 31.15 *dB*. The measured abrasive area was measured at 34.08 $\%$ at curvature 1, 35.26 $\%$ at curvature 2, and 56.33 $\%$ at curvature 3. As a result of comparing the sanded area of the traditional 2-point support mechanism and that of the 4-point support mechanism, it was confirmed that the sanding area was improved by 43 $\%$ in the 4-point support mechanism.

Because it has excellent curved surface adaptability, the adaptive belt sanding robot equipped with a 4-point support mechanism is expected to be applicable when sanding not only large objects with convex surfaces but also large objects with concave surfaces.

This proposed sanding robot can uniformly sand not only convex but also complex curved surfaces with various curvatures including concave target surfaces. These problems might be solved by active robot arms. We proposed a spring was mounted on the robot arm to generate a force in the direction of passive pushing with a spring force. In concave or complex curved surfaces with inconsistent curvature, the magnitude of the pushing force changes in real time depending on the shape of the target surface and must be precisely adjusted. The force generated by the robot arm must be actively changed to the desired value for complex curved surfaces. If an active robot arm is developed, the shape of the curved surface can be determined by measuring tension and surface pressure from the reaction force applied to the robot arm.

### Supplementary Information

Supplementary Information.

## Data Availability

All data generated or analyzed during this study are included in this published article and its Supplementary Information files.
